# Immune-related encephalitis after immune checkpoint inhibitor therapy

**DOI:** 10.1093/oncolo/oyae186

**Published:** 2024-07-26

**Authors:** Monica W Buckley, Aanika Balaji Warner, Julie Brahmer, Laura C Cappelli, William H Sharfman, Ephraim Fuchs, Hyunseok Kang, Patrick M Forde, Douglas E Gladstone, Richard Ambinder, Ronan J Kelly, Evan J Lipson, Ivana Gojo, Edward J Lee, Tory P Johnson, Shiv Saidha, Rafael Llinas, Lyle W Ostrow, Jarushka Naidoo, John C Probasco

**Affiliations:** Department of Neurology, Johns Hopkins University School of Medicine, Baltimore, MD 21287, United States; Department of Neurology, University of Virginia School of Medicine, Charlottesville, VA 22903, United States; Department of Oncology, Johns Hopkins University School of Medicine, Baltimore, MD 21287, United States; Bloomberg-Kimmel Institute for Cancer Immunotherapy, Sidney Kimmel Comprehensive Cancer Center at Johns Hopkins, Baltimore, MD 21287, United States; Department of Oncology, Johns Hopkins University School of Medicine, Baltimore, MD 21287, United States; Bloomberg-Kimmel Institute for Cancer Immunotherapy, Sidney Kimmel Comprehensive Cancer Center at Johns Hopkins, Baltimore, MD 21287, United States; Department of Oncology, Johns Hopkins University School of Medicine, Baltimore, MD 21287, United States; Department of Medicine, Johns Hopkins University School of Medicine, Baltimore, MD 21287, United States; Department of Oncology, Johns Hopkins University School of Medicine, Baltimore, MD 21287, United States; Bloomberg-Kimmel Institute for Cancer Immunotherapy, Sidney Kimmel Comprehensive Cancer Center at Johns Hopkins, Baltimore, MD 21287, United States; Department of Oncology, Johns Hopkins University School of Medicine, Baltimore, MD 21287, United States; Department of Oncology, Johns Hopkins University School of Medicine, Baltimore, MD 21287, United States; Department of Medicine, University of California, San Francisco, San Francisco, CA 94143, United States; Department of Oncology, Johns Hopkins University School of Medicine, Baltimore, MD 21287, United States; Bloomberg-Kimmel Institute for Cancer Immunotherapy, Sidney Kimmel Comprehensive Cancer Center at Johns Hopkins, Baltimore, MD 21287, United States; Department of Oncology, Johns Hopkins University School of Medicine, Baltimore, MD 21287, United States; R.J. Zuckerberg Cancer Center at Hofstra/Northwell Health, Lake Success, NY 11042, United States; Department of Oncology, Johns Hopkins University School of Medicine, Baltimore, MD 21287, United States; Department of Oncology, Johns Hopkins University School of Medicine, Baltimore, MD 21287, United States; Charles A. Sammons Cancer Center, Baylor University Medical Center, Dallas, TX 75246, United States; Department of Oncology, Johns Hopkins University School of Medicine, Baltimore, MD 21287, United States; Bloomberg-Kimmel Institute for Cancer Immunotherapy, Sidney Kimmel Comprehensive Cancer Center at Johns Hopkins, Baltimore, MD 21287, United States; Department of Oncology, Johns Hopkins University School of Medicine, Baltimore, MD 21287, United States; Maryland Oncology Hematology, Columbia, MD 21044, United States; Department of Neurology, Johns Hopkins University School of Medicine, Baltimore, MD 21287, United States; Department of Neurology, Johns Hopkins University School of Medicine, Baltimore, MD 21287, United States; Department of Neurology, Johns Hopkins University School of Medicine, Baltimore, MD 21287, United States; Department of Neurology, Johns Hopkins University School of Medicine, Baltimore, MD 21287, United States; Department of Neurology, Lewis Katz School of Medicine at Temple University, Philadelphia, PA 19140, United States; Department of Oncology, Johns Hopkins University School of Medicine, Baltimore, MD 21287, United States; Bloomberg-Kimmel Institute for Cancer Immunotherapy, Sidney Kimmel Comprehensive Cancer Center at Johns Hopkins, Baltimore, MD 21287, United States; Department of Oncology, Johns Hopkins Bayview Medical Center, Baltimore, MD 21224, United States; Department of Medicine, Beaumont Hospital Dublin and RCSI University of Health Sciences, Dublin, 9, Ireland; Department of Neurology, Johns Hopkins University School of Medicine, Baltimore, MD 21287, United States

**Keywords:** encephalitis, autoimmune, immune checkpoint, cancer

## Abstract

**Background:**

Immune checkpoint inhibitors (ICI) have revolutionized cancer treatment but can trigger immune-related encephalitis. We report one of the largest case series of patients with immune-related encephalitis and review of the literature.

**Methods:**

Retrospective series of patients with immune-related encephalitis and literature review.

**Results:**

Fourteen patients with cancer treated with ICI (50% combination therapy) developed immune-related encephalitis. Diagnostic testing revealed cerebral spinal fluid (CSF) lymphocytic pleocytosis (85%) and elevated protein (69%), abnormal brain magnetic resonance imaging(MRI) (33%) or brain FDG-PET (25%), electroencephalogram (EEG) abnormalities (30%), and autoantibodies (31%). Encephalitis treatment included: corticosteroids (86%), intravenous immunoglobulin (IVIg) (36%), plasmapheresis (7%), and rituximab (29%). There were no deaths and 12 patients had significant recovery, although long-term complications were observed. All patients discontinued ICI. Longitudinal follow-up demonstrated anti-cancer response to ICI at 3 months (85%) and 6 months post-ICI initiation (77%). A literature review identified 132 patients with immune-related encephalitis. Most were treated with PD-1 inhibitors (18% combination). Common abnormalities included elevated CSF protein (84%) or pleocytosis (77%), abnormal brain MRI (65%), or autoantibodies (47%). Nearly all were treated with corticosteroids, many required additional therapy with IVIg (26%) or rituximab (12%). Most patients had clinical improvement (81%) but a minority (10%) had a clinical relapse after completing corticosteroid taper. ICIs were resumed in 7 patients (5%), with relapse in 3.

**Conclusions and relevance:**

Immune-related encephalitis is treatable and improves with corticosteroids in most cases but may require additional immunosuppression. Re-emergence of encephalitis is rare and does not typically result in adverse outcomes, and this should be considered in neurological immune-related adverse event management guidelines.

## Teaser

Immune checkpoint inhibitors have revolutionized cancer treatment but can trigger immune-related encephalitis. This article reports one of the largest case series of patients with immune-related encephalitis and a review of the literature.

**Implications for practice**

Encephalitis due to immune checkpoint inhibitors (ICE) is treatable and improves with corticosteroids in most cases but may require additional immunosuppression. Re-emergence of this phenomenon is rare and does not typically result in adverse outcomes. While current guidelines recommend halting ICE after irEncephalitis, continued research is needed to evaluate whether patients may resume therapy.

## Introduction

Immune checkpoint inhibitors (ICI) are monoclonal antibodies targeting pathways involved in maintenance of immune tolerance. These include antibodies against programmed death-1 (PD-1), PD ligand-1, (PD-L1), and cytotoxic T-lymphocyte antigen-4 (CTLA-4).^1^ ICI improve outcomes in patients with a variety of cancers, including melanoma, non-small cell lung cancer (NSCLC), and renal cell carcinoma (RCC), both in metastatic and early-stage settings.^1-8^ Immune-related adverse events (irAEs) can affect any organ with higher incidences after combination.^1,9,10^ Neurological irAEs (nirAEs) affect 1.5% of patients treated with ICI with serious events occurring in 0.2%-0.8%. Serious nirAEs include encephalitis, meningitis, meningoencephalitis, myasthenia, myositis, and vasculitis.^11-13^

Recently, a definition of immune-related encephalitis as a nirAE was proposed including diagnosis of autoimmune encephalitis and clinical improvement/stabilization with immunomodulation or discontinuation of ICI.^13^ In 2016, experts proposed criteria for the diagnosis of autoimmune encephalitis, incorporating subacute memory deficits with supporting findings such as magnetic resonance imaging (MRI) brain changes, new onset seizures, and/or cerebral spinal fluid (CSF) pleocytosis.^14^ Autoimmune encephalitis varies clinically, but most commonly presents with subacute memory deficits, confusion, mood or behavioral changes, seizures, and/or movement disorders and imaging is highly variable.^14^ MRI brain can be normal, but may evolve with emergence of fluid-attenuated inversion recovery (FLAIR) or T2 sequence hyperintensities in the medial temporal lobes (typically bilateral in limbic encephalitis), gray matter, and white matter. Fluorodeoxyglucose (FDG)-PET may show hypermetabolism in the mesiotemporal regions (such as in limbic encephalitis) or hypometabolism such as in the visual cortex in anti-*N*-methyl-d-aspartate receptor (anti-NMDAR) encephalitis.^15^ Electroencephalogram (EEG) abnormalities include focal or generalized slow activity, epileptiform activity, fast super-imposed on slow activity (delta brush observed in anti-NMDAR encephalitis), or seizures. CSF abnormalities include pleocytosis, elevated protein, elevated IgG relative to serum, and neuronal autoantibodies.^14^ Autoimmune encephalitis can be associated with malignancy in the absence of ICI treatment.^14^

Here, we present a retrospective cohort of patients with immune-related encephalitis and review of the literature. We aim to determine whether patients fulfilled consensus criteria for autoimmune encephalitis, annotate management and response, and investigate long-term cancer and nirAE outcomes.

## Methods

We retrospectively identified patients hospitalized at the Johns Hopkins Hospital and Johns Hopkins Bayview Medical Center between June 1, 2014 and July 31, 2020 through a review of records and an institutional immune-related toxicity team consultation service.^16^ Patients and the public were not involved in the design, conduct, or reporting of this study. Included patients fulfilled 2016 consensus clinical criteria for diagnosis of possible, probable, or definite autoimmune encephalitis and had received at least 1 dose of ICI as their most recent cancer therapy with stabilization or clinical improvement after immunomodulation or discontinuation of ICI.^14^ Criteria for possible autoimmune encephalitis included the subacute onset of memory deficits, altered mental status, and/or psychiatric symptoms accompanied by at least one of the following: new focal neurological findings, seizures, CSF pleocytosis, and/or brain MRI suggestive of encephalitis with exclusion of alternative causes.^14^ Patients who met criteria for possible autoimmune encephalitis were further evaluated to determine whether they met criteria for probable or definite autoimmune encephalitis. Patients with neuronal cell-surface or onconeural autoantibodies detected in the serum and/or CSF or typical findings of limbic encephalitis with either CSF pleocytosis or epileptiform activity involving the temporal lobes were classified as having definite autoimmune encephalitis. Probable autoimmune encephalitis included patients who did not fulfill criteria for definite autoimmune encephalitis but with at least 2 of the 3 supportive findings: MRI suggestive of autoimmune encephalitis, CSF pleocytosis, presence of CSF-specific oligoclonal bands and/or elevated IgG-index, or brain biopsy showing inflammatory infiltrates.^14^ Board-certified neurologist independently adjudicated the diagnosis (J.P.). Patient demographics, presentation, diagnostic results, immunosuppressive treatments, neurological outcomes, and response to ICI were collected by chart review (M.B.; A.B., J.P.). First-line immunosuppressive treatments for autoimmune encephalitis included intravenous (IV) methylprednisolone or oral prednisone, intravenous immunoglobulin (IVIg), and plasmapheresis (PLEX), while rituximab and cyclophosphamide were considered second-line therapy.^12^ Antibody Prevalence in Epilepsy and Encephalopathy (APE^2^) and Responsive to Immunotherapy in Epilepsy and Encephalopathy (RITE^2^) scores were determined retrospectively (A.B., J.P.).^17^ Tumor response and durable clinical benefit to ICI were defined as a radiologic reduction in size or stable disease on contrast-enhanced CT imaging at 3 and 6 months post-ICI-initiation respectively and verified by an oncologist (J.N.). The severity of encephalitis for each patient was graded retrospectively per Common Terminology Criteria for Adverse Events (CTCAE) version 5.0.

A literature search for immune-related encephalitis was performed using PubMed, Embase, the Cochrane Library, Web of Science, Scopus, and ClinicalTrials.gov from respective database inception through October 2020. A search was constructed for encephalitis and ICI therapy (supplement). The search terms included controlled vocabulary, index terms, and additional keywords. Non-English language publications were excluded. There were 2601 records identified, 1134 duplicates removed and 1467 records screened. All abstracts were evaluated by independent reviewers (M.B., J.P.) using Covidence systematic review software (Veritas Health Innovation, Melbourne, Australia; www.covidence.org). Candidate studies were evaluated by full text (Supplementary Figure S1). Careful assessment ensured patients reported in different studies were excluded from duplicate analysis. A total of 85 individual publications and 132 patients were identified (Supplementary File S1). Each publication was reviewed to extract patient demographics and clinical data. Using available data, the patients were categorized as having a diagnosis of possible, probable, or definite autoimmune encephalitis as defined above.^14^

## Results

### Patients

We identified 14 patients with immune-related encephalitis (Table 1).^14^ The median age was 57.5 years (IQR: 27.3 years). Cancer diagnosis included: NSCLC (21%), melanoma (14%), small cell lung cancer (SCLC; 14%), Hodgkin’s lymphoma (14%), and other cancers (36%) (Table 1). Only 29% of patients had a history or suspicion of brain metastasis at presentation. Patients were treated with nivolumab (7%), pembrolizumab (36%), atezolizumab (7%), and combination therapy (50%) with primarily ipilimumab/nivolumab (Table 1). The time of onset of immune-related encephalitis after ICI initiation varied from 2 days to 71 weeks (median: 70 days, IQR 219.8 days) (Table 1).

**Table 1.** Patients with immune-related encephalitis after treatment with immune checkpoint inhibitors.

Pt. no.	Cancer	ICI regimen Time to onset	Symptoms on admission and CTCAE grade for ICI-related encephalitis	Other adverse events	APE^2^ score/RITE^2^ score	Intervention	Response Time to recovery (weeks)	Oncologic status at last follow up, total follow up after ICI-related encephalitis (weeks)
1	Melanoma, Brain metastasis	Nivo/ipi, 6 weeks	Headache, N/V, fevers Gait impairment Confusion, AMS, agitation requiring intubation R arm twitching CTCAE 3		8/10	IV MP 1g x 3 d IV MP 1g x 5 d Antibiotics, acyclovir Levetiracetam Steroid taper	Resolved 3 weeks	No Clinical Response: Deceased due to progressive cancer Total follow up ~15 weeks
2	SCC of skin	Pembro, 7 weeks	Headache Cognitive changes, AMS Fatigue, sleepiness CTCAE 3	Nephritis (simultaneous)	4/6	IV MP 1g x 5d Steroid taper	Resolved 6 weeks	Alive Clinical Response: Stable disease at 3 months post ICI No Durable Clinical Benefit: Progressive disease at 6 months post-ICI Total follow up ~216 weeks
3	Adnexal carcinoma of skin	Nivo/ipi, 22 weeks	Headache, N/V, fevers Confusion, memory impairment, personality changes Double vision, fatigue, dysarthria, CTCAE 3	Thyroiditis (antecedent) Hepatitis (antecedent) Myasthenia (simultaneous)	7/9	IV MP 1 g x 5d Antibiotics Long term steroids	Resolved 12 weeks	Clinical Response: Stable disease at 3 months post-ICI Durable Clinical Benefit: Stable disease at 6 months post-ICI Deceased due to progressive cancer Total follow up ~75 weeks
4	HNSCC	Nivo/ipi, 35 weeks	Headache, photophobia, N/V, chills, neck pain Mild confusion CTCAE 3		7/9	IV MP 1 g x 1d Steroid taper Acyclovir Relapse: IV MP 1 g x 3 days, steroid taper	Resolved 24 hours	Clinical Response: Clinical remission at 3 months post-ICI Durable Clinical Benefit: Clinical remission at 6 months post-ICI Clinical remission at 32 months post-AE Total follow up ~255 weeks
5	NSCLC	Pembro, 53 weeks	Cognitive decline, confusion Hallucinations, hyperactive delirium, picking behavior Gait impairment, falls CTCAE 3	Skin rash (subsequent) Hypophysitis (subsequent) PMR	5/7	Antibiotics, acyclovir 1 mg/kg IV MP, increased to 1g x 5 d Long term steroids	Resolved 4 weeks	Clinical Response: Clinical response at 3 months post-ICI Durable Clinical Benefit: Clinical response at 6 months post-ICI Alive with clinical response Total follow up ~154 weeks
6	Papillary Thyroid Ca	Pembro, 13 weeks	Mild confusion Ptosis, fatigue, diplopia CTCAE 3	Myasthenia (simultaneous)	5/5	IVIG x 5 d Long term IVIG	Resolved 5 days	Clinical Response: Stable disease at 3 months post-ICI Durable Clinical Benefit: Stable disease at 6 months post-ICI Alive with stable disease Total follow up ~264 weeks
7	NSCLC	Atezo, 2 days	Cognitive decline Gait impairment Bradykinesia, dysmetria Emotional lability CTCAE 3	Immune Thrombo-cytopenia (simultaneous)	6/8	80 mg prednisone IV MP 1 g x 5 d IVIG x 5 d Steroid taper	Minimal improvement while hospitalized	Lost to follow-up post-hospitalization Total follow up ~1 week
8	Hodgkin Lymphoma	Nivo/anti-LAG3, 42 weeks	Hallucinations, delusions, psychosis, pressured speech, paranoia, grandeur CTCAE 4		6/8	IV MP 1 g x 5 d PLEX x 5 d Rituximab	Resolved 4 weeks	Clinical Response: Clinical response at 3 months post-ICI No Durable Clinical Benefit: Clinical progression at 6 months post-ICI Alive following treatment for progression Total follow up ~207 weeks
9	Hodgkin/ Grayzone Lymphoma	Nivo, 27 weeks	Seizures Headaches, nausea Memory loss Difficulty with concentration Aphasia, dysarthria Personality changes, irritable, easy to anger CTCAE 3	Thyroiditis (antecedent)	12/14	IV MP 1g x 5 d Acyclovir Levetiracetam, lacosamide, clobazam IVIG x 5 d Rituximab Corticosteroid taper Relapse: IV MP 1g x 5 d	Resolved 6 weeks	Clinical Response: Clinical response at 3 months post-ICI Durable Clinical Benefit: Clinical response at 6 months post ICI Alive with clinical remission Total follow up ~171 weeks
10	SCLC, Brain metastasis	Nivo/ipi, 3 days	Memory loss Gait impairment Tremor, ataxia CTCAE 3	SIADH (simultaneous)	7/9	Prednisone 60 mg Steroid taper	Resolved 4 weeks	Clinical Response: Clinical response at 3 months post-ICI Durable Clinical Benefit: Clinical response at 6 months post-ICI Deceased due to progressive disease Total follow up ~33 weeks
11	Melanoma, Brain metastasis	Nivo/ipi, 15 days	Fevers, N/V Memory loss, AMS Inappropriate laughter Gait impairment Dysautonomia Seizures CTCAE 3		10/14	IV MP 1 g x 5 d IVIG Rituximab Levetiracetam, phenytoin, lacosamide	Resolved 12 weeks	Clinical Response: Clinical response at 3 months post-ICI Durable Clinical Benefit: Clinical response at 6 months post-ICI Deceased due to progressive disease Total follow up ~160 weeks
12	SCLC	Nivo/ipi 10 days	Confusion Headache, N/V, chills Gait impairment, falls Ataxia CTCAE 3	SIADH	7/9	IV MP 0.5 g x 4 d Steroid taper	Resolved 4 weeks	Clinical Response Clinical response at 3 months post-ICI Durable Clinical Benefit: Clinical response at 6 months post-ICI Alive with sustained response Total follow up ~372 weeks
13	AML	Pembro, 9 days	AMS CTCAE 3	Dermatitis	9/9	None	Resolved 6 weeks	Clinical response at 3 months post-ICI Durable Clinical Benefit: Clinical response at 6 months post-ICI Deceased due to progressive disease Total follow up ~29 weeks
14	NSCLC, suspected brain metastasis	Pembro, 71 weeks	AMS, seizures, aphasia, vertigo CTCAE 4		7/9	IV MP 1 g x 5 d IVIG Rituximab Prednisone taper	No improvement at 3 months post-AE onset	Clinical response at 3 months post-ICI Durable Clinical Benefit: Clinical response at 6 months post-ICI Lost to follow-up after entering hospice Total follow up ~12 weeks

Abbreviations: AML, acute myelogenous leukemia; AMS, altered mental status; APE^2^, antibody prevalence in epilepsy and encephalopathy; Atezo, atezolizumab; CTCAE, common terminology criteria for adverse events; d, days; F, female; HNSCC, head and neck squamous cell carcinoma; IVIG, intravenous immunoglobulins; IV, intravenous; M, male; MP, methylprednisolone; N/V, nausea and vomiting; nivo, nivolumab; ipi, ipilimumab; NSCLC, non-small cell lung cancer; No., number; Pt., patient; pembro, pembrolizumab; RITE^2^, response to immunotherapy in epilepsy and encephalopathy; SCC, squamous cell carcinoma; SCLC, small cell lung cancer; SIADH, syndrome of inappropriate antidiuretic hormone secretion.

The literature review identified 132 patients with immune-related encephalitis. Median age was 61.5 years (IQR: 17 years). Patients had melanoma (32%), NSCLC (26%), RCC (8%), and hematologic malignancies (5%). Brain metastasis was present in 20% of patients. Most patients were treated with PD-1 inhibitors (63%) and approximately 18% of patients were treated with combination ICI (Supplementary Table S1). The onset of symptoms from initiation of ICI ranged from less than 24 hours to more than 1 year with a median of 62 days (IQR 110.75 days; Supplementary Table S1).

### Clinical presentation

Patients in the institutional cohort presented with confusion, memory impairment, and/or altered mental status 93%; gait imbalance (43%); definite or suspected seizures (29%); fevers/chills (21%); meningeal signs reflecting meningeal inflammation or meningitis including headache (50%) and personality changes (29%; Table 1). Thirteen patients presented with a combination of symptoms, most commonly headache with altered mental status (43%) reflective of likely meningoencephalitis. Most patients (64%) were diagnosed with additional irAEs (Table 1) including 2 patients with myasthenia. Due to the presence of concurrent irAEs (14%) and treatment for brain metastasis (7%), 3 patients were on corticosteroids at the time of developing symptoms of immune-related encephalitis. The severity of encephalitis was as follows: grade 3 (86%) and grade 4 (14%; Table 1).

Among the patients identified in the literature review, 92% met the criteria for autoimmune encephalitis based on available data: possible (51%), probable 4%, and definite (37%; Supplementary Table S1). Typical symptoms included: confusion, altered mental status, or memory impairment (87%) and psychiatric symptoms (25%). Signs of meningeal inflammation were common and included fevers (28%), headaches (21%), and nausea/vomiting (15%) (Supplementary Table S2). Approximately 27% of patients presented with seizures. Interestingly, concurrent irAEs were common (33%), and included dermatitis (10%), hepatitis (6%), hypophysitis (2%), and thyroiditis (5%). Concurrent nirAEs occurred in 17 patients (13%) and included sensory neuronopathy (3%), optic neuritis (2%), Guillain-Barre syndrome (1.5%) and myopathy (1.5%) (Supplementary Table S2).

### Diagnostic evaluation

**Figure 1.**
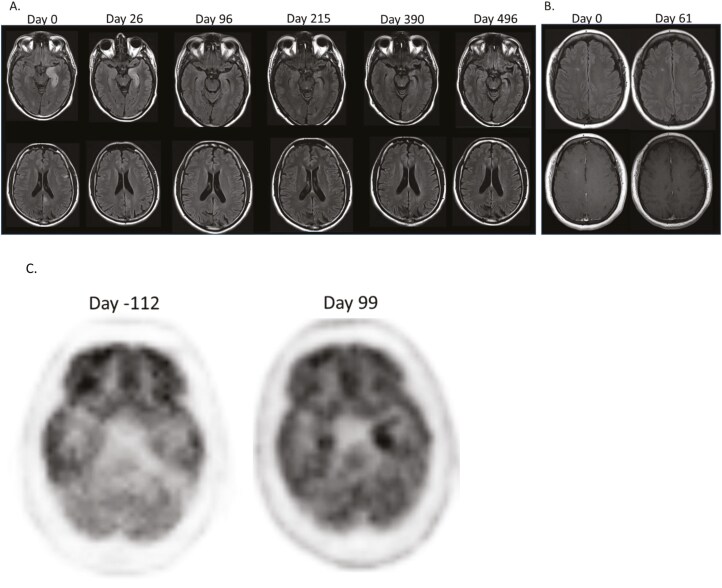
MRI and brain FDG-PET imaging of patients with immune-related encephalitis. (A) T2/FLAIR MRI brain images at presentation and follow up after treatment in a patient with typical imaging findings of autoimmune encephalitis (patient 9). Note T2/FLAIR hyperintensities of the left hippocampus, left frontal lobe, and posterior cingulate. The left hippocampal lesion evolves to medial temporal sclerosis while the left frontal and posterior cingulate lesions resolve. (B) T2 FLAIR and T1-post-gadolinium MRI brain images at presentation and after treatment in patient with lesion of the right corona radiate demonstrating persistence of T2/FLAIR hyperintensity with resolution of gadolinium enhancement on follow-up (patient 6). (C) FDG-PET/CT brain imaging 112 days prior to and 99 days after immunosuppressive treatment initiation for immune-related encephalitis demonstrating hypermetabolism of the left medial temporal lobe that was not evident on the pre-encephalitis FDG-PET performed through the course of oncologic care (patient 9).

Among the patients in the institutional cohort, the differential diagnoses included brain metastasis, leptomeningeal disease, infectious meningitis, or infectious encephalitis. The full evaluation included testing for neuronal autoantibodies (*n* = 13), brain MRI (*n* = 14), lumbar puncture (*n* = 13), brain FDG-PET/CT (*n* = 4), EEG (*n* = 10), and electromyogram and nerve conduction studies (EMG/NCS; *n* = 2) (Table 2). Brain imaging was unremarkable or non-specific in the majority of patients (64%). Two patients had image findings typical of autoimmune encephalitis and one patient had a single contrast-enhancing lesion (Table 2; Figure 1A, B). On brain FDG-PET/CT imaging, 1 patient had findings consistent with autoimmune encephalitis with hypermetabolism in the medial temporal lobe (Figure 1C). CSF analysis frequently revealed lymphocytic pleocytosis (85%) and/or elevated protein levels (69%). All patients had negative infectious CSF evaluations. Oligoclonal bands were evaluated in 10 patients with 60% having matched oligoclonal bands in their serum and CSF and 2 patients with unique oligoclonal bands in the CSF alone, consistent with intrathecal immunoglobulin synthesis. Only 3 patients (30%) had epileptiform activity or seizures on EEG. All 14 patients had an APE^2^ score ≥4, suggestive of an associated neuronal autoantibody. Paraneoplastic/autoimmune encephalopathy autoantibody testing was completed in 12 patients (*n* = 3 serum, *n* = 5 CSF, *n* = 4 both); 25% had positive testing on commercially available tests (anti-NMDAR, AGNA, VGKC complex without further specification) while one patient had a positive autoantibody on research testing (anti-neurofilament light chain; Table 2). Five patients (36%) met the criteria for definite autoimmune encephalitis based on the presence of autoantibodies or typical imaging findings.

**Table 2.** Evaluation of cohort patients with immune-related encephalitis.

	MRI brain	Lumbar puncture			Autoantibodies	EEG
		**Cell count** **(cells/µL)**	**Protein** **(mg/dL)**	**Oligoclonal bands (OCBs)**		
1	Hemorrhagic metastasis	166	80	ND	Negative	Generalized periodic discharges
2	WNL	0	19.7	Pattern 4, OCBs in CSF identical to those in serum	Negative	WNL
3	WNL	12	53.4	None	Negative	WNL
4	WNL	75	68	ND	ND	ND
5	Right Parietal infarction	12	39	ND	Negative	Slow
6	T2/FLAIR in left corona radiata with contrast enhancement	ND	ND	ND	AChR Ab+	ND
7	WNL	1	53.2	None	Negative	WNL
8	WNL	20	113.1	Pattern 4, OCBs in CSF identical to those in serum	Negative	WNL
9	T2/FLAIR hyperintensities of left medial temporal lobe, left lateral frontal lobe and posterior cingulate	22	37.7	Pattern 4, OCBs in CSF identical to those in serum	Negative	Left temporal spikes and slowing
10	Right hippocampus T2/FLAIR hyperintensity	18	98	Pattern 4, OCBs in CSF identical to those in serum	Anti-glial nuclear Ab	ND
11	Metastatic disease	8	24	Pattern 4, OCBs in CSF identical to those in serum	NMDAR Ab	Left temporal spikes and seizures
12	Nonspecific subcortical T2/FLAIR lesions	30	166	Pattern 4, OCBs in CSF identical to those in serum	Novel unclassified Ab subsequently identified as directed to neurofilament light chain	WNL
13	Multiple cortical and subcortical T2/FLAIR hyperintensities, some of which enhance	8	64.6	Pattern 2 with ≥2 OCBs in CSF only	Negative	ND
14	Punctate enhancing T2/FLAIR hyperintensities concerning for metastases as well as nonspecific subcortical T2/FLAIR hyperintensities	6	68	Pattern 3, ≥2 OCBs in CSF with ≥1 separate bands in CSF also in serum	VGKC complex antibody, LGI1 and CASPR2 negative	Diffuse, symmetric irregular occipital rhythm and background slowing without epileptiform activity

Abbreviations: Ab, antibody; AChR, Acetylcholine Receptor; CASPR2, contactin-associated protein-like 2; EEG, electroencephalogram; FLAIR, fluid-attenuated inversion recovery; LGI1, leucine-rich glioma inactivated 1; MRI, magnetic resonance imaging; ND, not done; NMDA-R, N-methyl-D aspartate receptor; OCBs, oligoclonal bands; VGKC, voltage gated potassium channel; WNL, within normal limits.

Among patients identified in the systematic review, evaluations typically included brain MRI, lumbar puncture, and EEG. MRI brain imaging was unremarkable or stable in 35% of patients. Typical findings such as bilateral temporal or multiple T2/FLAIR lesions were frequently observed (21% and 19%, respectively). Leptomeningeal or dural enhancement was seen primarily in patients presenting with CSF pleocytosis and meningeal symptoms suggestive of meningoencephalitis (Table 3). Of note, several patients initially had unremarkable brain MRI, but had abnormal follow-up imaging (*n* = 11) with findings of autoimmune or limbic encephalitis, dural/leptomeningeal enhancement, or demyelination.

**Table 3.** Summary of evaluation of patients with immune-related encephalitis as a neurological immune related adverse event following immune checkpoint inhibitor therapy identified by systematic literature review.

	Lumbar Puncture (103/132)				MRI brain (113/132)						Auto- Antibodies (96/132)	
	Cell Count (cells/ul) (103/132)		Protein (mg/dl) (89/132)									
	Normal (24/103)	Abnormal (79/103)	Normal (15/89)	Abnormal (74/89)	Stable WNL	Typical LE	Typical AE	Demye-llinating	Enhancement (lepto/dural)	Non-specific	Negative	Positive
Total no. (%)	24 (23.3%)	79 (76.7%)	15 (16.9%)	74 (83.1%)	40 (35.4%)	24 (21.2%)	21 (18.6%)	6 (5.3%)	13 (11.5%)	9 (7.9%)	51 (56.1%)	45 (46.9%)
Mean (SD)		69.2 (115.3)		183.8 (251.7)								

Abbreviations: AE, autoimmune encephalitis; LE, limbic encephalitis; MRI, magnetic resonance imaging; WNL, within normal limits.

CSF analysis included cell count, protein, glucose, infectious studies, cytopathology or flow cytometry, and autoantibody testing. CSF pleocytosis was common, although 23% of patients had normal CSF white blood count (WBC). Lymphocytic pleocytosis was predominant and observed in 93% of patients with pleocytosis. However, neutrophilic pleocytosis was seen (7%). Most patients had elevated CSF protein (84%, Table 3). Infectious workup and additional screening for malignancy with CSF cytopathology or flow cytometry was negative in all cases tested.

A total of 96 patients were tested for specific neuronal autoantibodies, of these 45 patients (47%; Table 3) had positive results. Detected autoantibodies primarily targeted intracellular antigens and included: anti-Hu (16%), anti-Ma2 (22%), anti-GAD65 (11%), anti-GFAP (9%), and a neuron specific autoantibody of unknown specificity (18%). Anti-NMDAR encephalitis was diagnosed in 4 patients (80%).^18^ Seven patients (16%) had more than 1 autoantibody identified. Retrospective analysis of pre-ICI serum revealed the presence of autoantibodies (*n* = 5); however, pre-treatment samples were rarely tested (numbers unavailable). In some cases, patients had other classical paraneoplastic syndromes associated with the detected autoantibody; for example, sensory neuronopathy was diagnosed in a patient with anti-Hu autoantibody.^19,20^

EEG results were reported in 47 patients (36%) and included slowing or consistent with diffuse encephalopathy (64%) and slow activity or temporal lobe epileptiform activity (13%) (Table 3). Confirmed seizures were captured in 4 patients and 2 patients presented with status epilepticus.

### Treatment of immune-related encephalitis

Among the institutional cohort, clinical suspicion for immune-related encephalitis resulted in discontinuation of ICI and initiation of immunosuppression in 13 cases. Given subacute presentation, 1 patient received additional ICI after initial symptoms (patient 5). RITE^2^ score was ≥7 in 12 patients (86%), predictive of a favorable response to immunosuppression. Intravenous corticosteroids (500 or 1000 mg IV methylprednisolone) for 3-5 days with corticosteroid taper was the typical initial therapy, and 86% received corticosteroids. Treatment with IVIg was initiated in 36% of patients (*n* = 1 as initial therapy, *n* = 4 following IVMP) and plasmapheresis in one patient (7%). Patients who did not respond to first-line immunosuppressive therapy were treated with rituximab as second-line therapy (4/14, 29%; Table 1).

Most patients recovered from immune-related encephalitis over several weeks, although one patient was lost to follow up. Among the 13 with follow-up, the tempo of recovery was variable, and 1 patient (patient 4) with primarily meningeal symptoms with headaches and mild cognitive changes recovered immediately after corticosteroid initiation. Unfortunately, 1 patient did not demonstrate improvement after immunosuppression with rituximab (patient 14). As expected, patients with longer recovery time had more severe immune-related encephalitis characterized by seizures and cognitive impairment. Although most patients with follow-up had a marked clinical recovery (85%) and there were no cases of mortality due to ICI-related encephalitis, some patients had residual cognitive impairment (31%), recurrent seizures (8%), or required continued treatment with anti-seizure medications (23%) (Table 1). Two patients (15%) had a clinical relapse during corticosteroid taper which responded to repeat treatment with IV methylprednisolone.

Of patients in the literature review, ICI was discontinued in all patients with immune-related encephalitis, although 7 patients had repeat exposure after recovery for further cancer treatment. Patients received corticosteroids as first-line treatment, typically either IV methylprednisolone (500-1000 mg/day) or IV dexamethasone (1-2 mg/kg/day) for 3-5 days (Table 4). While improvement was seen with corticosteroids alone, patients frequently received additional first-line immunosuppressive therapy with IVIg (26%) and plasmapheresis (10%). The most common second-line therapy was rituximab (12%). No immunosuppressive medications were administered to 5% of patients due to unrecognized diagnoses or rapidly fatal or resolving symptoms. Patients with suspected or confirmed seizures were treated with anti-epileptics.

**Table 4.** Treatments and outcomes of reported patients with immune-related encephalitis as a neurological immune related adverse event following immune checkpoint inhibitor therapy identified by systematic literature review.

	PDL-1 inhibitors (atezolizumab)	PD-1 inhibitors (nivolumab/ pembrolizumab)	CTLA-4 inhibitors (ipilimumab)	Combination	All
**Treatment**					
IV or PO steroids	15	76	8	21	120 (96%)
IVIG	6	18	2	7	33 (26.4%)
Plasmapheresis	0	10	1	1	12 (9.6%)
Rituximab	3	8	0	4	15 (12%)
Other IS	3	8	2	1	14 (11.2%)
No IS	0	4	0	3	7 (5.6%)
**Outcome**					
Deceased from AE	0	11	0	3	
Time to recovery, days, mean (SD)	11.3(16.6)	18.7 (16.0)	31.8 (39.6)	18.2 (18.1)	18 (18.6)
Time to recovery, days, median (IQR)	5 (6.5)	14 (21.8)	17.5 (27.3)	8 (21)	9 (23)
Total deceased at follow up	4	24	0	9	37 (29.6%)
Time to follow up, days, mean (SD)	107.7 (94.6)	311.6 (391)	202.5 (51.2)	283.7 (361)	274.4 (354.7)
Time to follow up, days, median (IQR)	90 (45.3)	180 (305)	195 (52.5)	150 (127.5)	150 (245.5)

Abbreviations: AE, autoimmune encephalitis; IS, immunosuppressive treatment; IV, intravenous; IVIG, IV immunoglobulins; PO, per oral

Most patients had partial or complete recovery (81%). Typical findings of limbic encephalitis were more commonly found in patients with no recovery or death (82%) as compared to patients with partial or full recovery (25%). Additionally, patients with partial or full recovery were more likely to have a normal or stable MRI (54%). Stabilization of symptoms but minimal or no clinical improvement was associated with hippocampal atrophy secondary to inflammatory damage. Mortality without any recovery from immune-related encephalitis (12%) was attributed to encephalitis; however, progressive cancer, comorbid infections, and respiratory failure also contributed. Among patients who recovered, clinically significant improvement occurred on average 18 days after treatment and improvement continued over weeks. However, some patients with predominately meningeal symptoms had rapid recovery within 24 hours.

Relapse of immune-related encephalitis was infrequent (10%) and occurred during corticosteroid taper or re-exposure to ICI. Of the 7 patients re-exposed to ICI, 3 had relapse of encephalitis. Treatment for relapse was similar to initial treatment; corticosteroids were administered to all patients. However, 2 patients required treatment with rituximab and 1 patient died.

### Response to ICI in patients with immune-related encephalitis

Of patients in the institutional cohort with longitudinal follow-up (*n* = 13), 85% demonstrated anti-tumor response to ICI at 3-month post-ICI initiation, and 77% at 6-month follow-up (Table 1). Of those with a RITE^2^ score greater than or equal to 7 (*n* = 13), 85% had favorable responses to immunosuppression. At last follow-up, 38% of patients died of progressive disease, and 7 had stable disease or continued partial response (median follow up 1098 days, IQR 1290 days). Chronic corticosteroid use was in 38% for continued treatment for encephalitis, metastatic cancer, or hypophysitis. No patients were re-exposed to ICIs.

The patients identified in the systematic review were followed an average of 274 days after diagnosis of immune-related encephalitis (Table 4). After removing patients without available data, a total of 41 patients were reported to have died (33%). Cause of death included progressive malignancy (44%), immune-related encephalitis (37%), other irAEs such as autoimmune colitis (5%), or other comorbidities (12%).

## Discussion

Immune-related encephalitis is a well-recognized neurological irAE following ICI.^11,12^ Here, we present a retrospective 14 patient case series, one of the largest to date, accompanied by a review of the literature.

Melanoma and NSCLC were the most common underlying malignancies, likely reflecting the frequency of administration and length of time since FDA approval of ICI rather than intrinsic cancer factors as these malignancies are not frequently associated with paraneoplastic encephalitis.^1^ Most previously reported patients were treated with PD-1 inhibitors alone. However, our case series had a higher frequency of patients treated with combination ICI (44% as compared to 18% in the literature review). Additionally, brain metastasis were found in the minority of patients both in the case series as well as literature review (20%-30%). The presence of brain metastasis and treatment with radiation or radiosurgery may contribute to blood brain barrier breakdown and altered immune responses in the brain. Interestingly, although most irAE occur in the first 3 months of treatment, immune-related encephalitis could occur more than 1 year after first exposure, as is observed in pneumonitis.^21^ However, the onset is highly variable and symptoms can occur within 24 hours after the start of ICI.

Patients with immune-related encephalitis typically present with subacute cognitive changes, meningeal signs, and personality changes, similar to what is seen in autoimmune encephalitis cases not associated with ICI. These symptoms are also present in leptomeningeal carcinomatosis as well as infectious meningitis/encephalitis, highlighting the importance of excluding alternative diagnoses. Many patients had other co-existing irAEs, including nirAEs, reminding us that patients are at risk for multiple as well as isolated irAEs. Our series included 2 cases of encephalitis and myasthenia gravis, which to our knowledge has not been previously reported.

Interestingly, all patients within the institutional cohort had APE^2^ scores of ≥4 and 86% of the patients had a RITE^2^ score ≥7, predicting presence of a neuronal autoantibody and response of symptoms of encephalitis to immunotherapy. These scores may prove helpful in the identification of patients with immune-related encephalitis, guide considerations for systemic immunosuppression, and identify patients likely to have a novel neuronal autoantibody. Future prospective evaluation is warranted to assess the utility of these scores in the identification and response to immunosuppression among patients suspected of having immune-related encephalitis.

The typical workup of patients in both our institutional series and those identified through the literature review included an MRI brain, lumbar puncture, and EEG. One discrepancy included the frequency of patients with MRI brain findings typical of autoimmune encephalitis; 28% of our case series compared to over 50% in a literature review. This potentially reflects increased awareness allowing for diagnosis prior to MRI brain changes as untreated patients can develop MRI abnormalities over time. Lumbar puncture most commonly showed lymphocytic pleocytosis or elevated protein. Workup included evaluation for infectious etiologies, and none of our patients with concern for immune-related encephalitis had. Nearly half of patients in the literature review who were tested for neuronal autoantibodies had positive titers. Although this may reflect publication bias, a recent study revealed a high frequency of neuronal autoantibodies in patients with immune-related encephalitis.^22^ Importantly, patients with neuronal autoantibodies often had normal MRI brain and lumbar puncture, highlighting that normal studies do not rule out immune-related encephalitis and clinical suspicion is paramount.

In our case series, patients commonly responded to first-line immunosuppressive therapy defined as corticosteroids, IVIg, or plasmapheresis and had lower mortality than the literature cohort. However, recovery was variable with some patients having nearly immediate improvement after initiation of corticosteroids while other patients died or had no recovery from their autoimmune encephalitis. Associated factors included abnormal MRI brain, especially typical findings of limbic encephalitis which was found in 82% of patients with poor recovery or death and only 25% of patients with partial/full recovery. Although CSF pleocytosis was not predictive of recovery in our literature review cohort, neuronal autoantibodies were found in 62% of patients with poor recovery or death and only 38% of patients with partial/full recovery. In both cohorts, the relapse rate was lower than typical autoimmune encephalitis, suggesting that long-term immunosuppressive therapy may not be required. Close clinical monitoring should be continued during corticosteroid taper as some patients relapsed during corticosteroid discontinuation, although corticosteroid taper should be decided on a case by case basis.^23,24^ Considering that patients in our cohort responded well to immunosuppression raises the question of whether ICI could be continued if clinically indicated. It may be that patients can be treated through the acute encephalitis and further ICI may be administered with careful neurological monitoring and rapid re-initiation of immunosuppressive treatments with recurrence.

Further research is required in this burgeoning area of nirAEs. It is not known whether ICI therapy could unmask latent disease. Some patients had neuronal autoantibodies identified in pre-treatment serum without a neurological syndrome. Although this was infrequently tested. It is difficult to determine how many cases of immune-related encephalitis represent pre-existing paraneoplastic encephalitis. Recent studies have shown that pre-existing paraneoplastic disorders have worsened symptoms in up to 50% of patients after ICI therapy.^25^ Identification of biomarkers will be crucial for diagnosis of pre-existing paraneoplastic neurological syndromes, diagnosis of nirAEs, and monitoring response of nirAEs to immunosuppressive therapy.^11^ Finally, most patients with immune-related encephalitis in this case series and literature review responded well to immunotherapy. Continued research is needed to evaluate whether the current guidelines that recommend halting ICI therapy after immune-related encephalitis should be revised.

## Supplementary Material

oyae186_suppl_Supplementary_Tables_Figure

oyae186_suppl_Supplementary_File_S1

## Data Availability

The data underlying this article will be shared on reasonable request to the corresponding author.
