# Supplementary material for: Immune-related encephalitis after immune checkpoint inhibitor therapy
Source: Oncologist. 2024 Jul 26;30(1):oyae186. doi: 10.1093/oncolo/oyae186 (PMC11783331; doi:10.1093/oncolo/oyae186)
Supplement: oyae186_suppl_Supplementary_Tables_Figure [file oyae186_suppl_supplementary_tables_figure.docx]

**Supplementary Figure 1: CONSORT diagram of literature review**

**
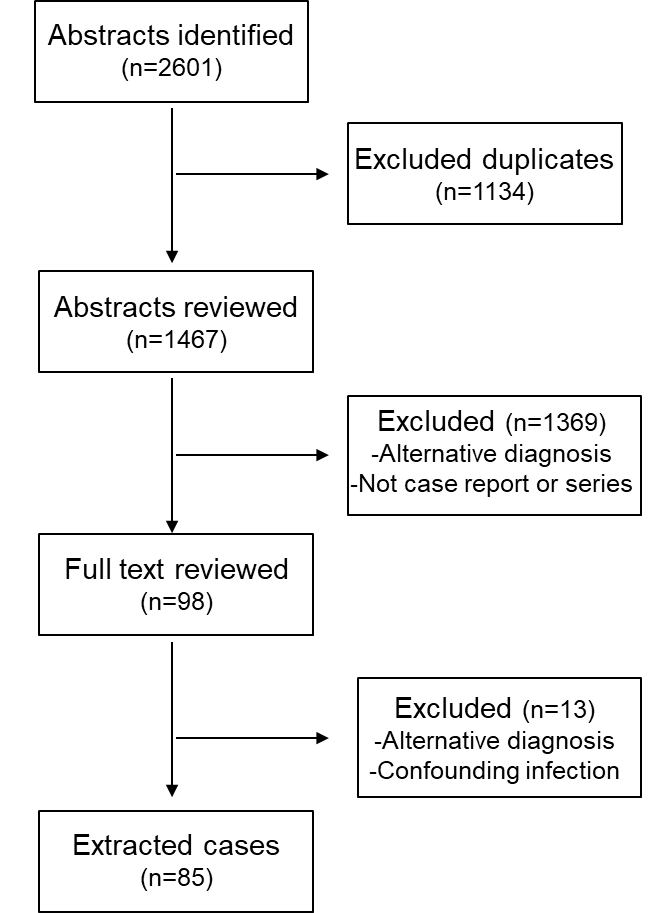
**

**Supplementary Table 1: Patient demographics, treatment, and outcomes in patients treated with different immune checkpoint inhibitors who developed immune-related encephalitis identified by systematic literature review.**

Abbreviations: AE, autoimmune encephalitis; CTLA4, cytotoxic lymphocyte-associated antigen 4; ICI, immune checkpoint inhibitor; IPI, ipilimumab; NIVO, nivolumab; NSCLC, non-small cell lung cancer; Pembro, pembrolizumab; SCLC, small cell lung cancer, SD, standard deviation

|  | **PD-L1 inhibitors (atezolizumab)** | **PD-1 inhibitors (nivolumab, pembrolizumab)** | **CTLA4 inhibitors**  **(ipilimumab)** | **Combination** | **All** |
| --- | --- | --- | --- | --- | --- |
| **Demographics** |  |  |  |  |  |
| Number of patients (%) | 15(7.6%) | 83(62.9%) | 8 (6.1%) | 24 (18.2%) | 132 (100%) |
| Age, years, mean (SD) | 58.3 (12.1) | 59.7 (16.5) | 49.6 (11.2) | 59.4 (13.1) | 58.9 (15.1) |
| Age, years, median (range) | 59 (37-78) | 65 (9-83) | 45 (39-67) | 61.5 (19-76) | 61.5 (9-83) |
| **Malignancy** |  |  |  |  |  |
| Melanoma | 0 | 18 | 7 | 16 | 42 (31.8%) |
| SCLC | 2 | 4 | 0 | 3 | 9 (6.8%) |
| NSCLC | 3 | 30 | 0 | 0 | 34(25.8%) |
| Renal | 0 | 8 | 0 | 2 | 10 (7.6%) |
| Hodgkin | 0 | 7 | 0 | 0 | 7 (5.3%) |
| Other | 10 | 16 | 1 | 3 | 30 (22.7%) |
| Brain Metastasis | 1 | 18 | 2 | 5 | 26 (19.7%) |
| **ICI duration** |  |  |  |  |  |
| Onset from initial cycle, days, mean | 30 (48.6) | 121.4 (143.4) | 141.4 (174.2) | 91.2 (92) | 107.4 (130.4) |
| Onset from initial cycle, days, median | 13 (6-150) | 77 (1-630) | 61 (42-450) | 65.5 (4-300) | 62 (1-630) |
| **Diagnosis** |  |  |  |  |  |
| Possible AE | 10 | 37 | 5 | 15 | 67 (50.8%) |
| Probable AE | 0 | 3 | 1 | 1 | 5 (3.8%) |
| Definite AE | 4 | 35 | 1 | 7 | 49 (37.1%) |
| Unlikely | 1 | 8 | 1 | 1 | 11 (8.3%) |

**Supplementary Table 2: Reported symptoms of reported patients with immune-related encephalitis as a neurologic immune related adverse event following immune checkpoint inhibitor therapy identified by systematic literature review.**

| **Symptom** | **Number of patients (%)** |
| --- | --- |
| Confusion | 104 (87.4%) |
| Seizures | 32 (26.9%) |
| Headaches | 25 (21.0%) |
| Fevers/Chills | 33 (27.7%) |
| Nausea/vomiting | 18 (15.1%) |
| Psychiatric/Personality changes | 30 (25.2%) |
| Gait imbalance | 25 (21.0%) |
| Ataxia | 25 (21.0%) |
| Aphasia | 20 (16.8%) |
| Abnormal movements/Movement disorders | 22 (18.5%) |
| Other adverse events | 39 (32.8%) |
| Other neurologic adverse events | 17 (14.3%) |
