# Supplementary material for: Immune-related encephalitis after immune checkpoint inhibitor therapy
Source: Oncologist. 2024 Jul 26;30(1):oyae186. doi: 10.1093/oncolo/oyae186 (PMC11783331; doi:10.1093/oncolo/oyae186)
Supplement: oyae186_suppl_Supplementary_File_S1 [file oyae186_suppl_supplementary_file_s1.docx]

**Supplement: Case series included in literature review.**

1. Arakawa M, Yamazaki M, Toda Y, Saito R, Ozawa A, Kosaihira S, et al. Atezolizumab-induced encephalitis in metastatic lung cancer: a case report and literature review. eNeurologicalSci. 2019. 14:49-50. doi: 10.1016/j.ensci.2018.12.001.
2. Boekstegers AM, Blaeschke F, Schmid I, Wiebking V, Immler S, Hoffmann F, et al. MRD response in a refractory paediatric T-ALL patient through anti-programmed cell death 1 (PD-1) Ab treatment associated with induction of fatal GvHD. Bone Marrow Transplant. 2017. 52(8):1221-1224. doi: 10.1038/bmt.2017.107.
3. Bossart S, Thurneysen S, Rushing E, Frontzek K, Leske H, Mihic‐Probst D, et al. Case Report: Encephalitis, with Brainstem Involvement, Following Checkpoint Inhibitor Therapy in Metastatic Melanoma. Oncologist. 2017. 22(6):749-753. doi: 10.1634/theoncologist.2016-0366.
4. Bross SP, Mongelluzzo GJ, Conger AR, Patel MA, Vadakara J, Grant M, et al. Case Report of Immuno-Oncotherapy (IO) Provoked Encephalitis Mimicking Brain Metastasis in a Patient with History of Traumatic Brain Injury. World Neurosurg. 2020;139:483–7. doi: 10.1016/j.wneu.2020.04.177.
5. Brown MP, Hissaria P, Hsieh AH, Kneebone C, Vallat W. Autoimmune limbic encephalitis with anti-contactin-associated protein-like 2 antibody secondary to pembrolizumab therapy. J Neuroimmunol. 2017. 305:16-18. doi: 10.1016/j.jneuroim.2016.12.016.
6. Burke M, Hardesty M, Downs W. A case of severe encephalitis while on PD-1 immunotherapy for recurrent clear cell ovarian cancer. Gynecol Oncol Reports. 2018. 24:51-53. doi: 10.1016/j.gore.2018.03.007.
7. Cabral G, Ladeira F, Gil N. Nivolumab-induced seronegative encephalitis. J Neuroimmunol. 2020. 347:577350.  doi: 10.1016/j.jneuroim.2020.577350.
8. Carl D, Grüllich C, Hering S, Schabet M. Steroid responsive encephalopathy associated with autoimmune thyroiditis following ipilimumab therapy: A case report. BMC Res Notes. 2015. 8:316. doi: 10.1186/s13104-015-1283-9.
9. Chang H, Shin YW, Keam B, Kim M, Im SA, Lee ST. HLA-B27 association of autoimmune encephalitis induced by PD-L1 inhibitor. Annals of Clinical and translational neurology. 2020. 7(11):2243-2250. doi: 10.1002/acn3.51213.
10. Conry RM, Sullivan JC, Nabors LB. Ipilimumab-induced encephalopathy with a reversible splenial lesion. Cancer Immunol Res. 2015. 3(6):598-601. doi: 10.1158/2326-6066.CIR-15-0035.
11. Cooper J, Kodali D, Higa GM. Acute encephalopathy with combination dabrafenib/trametinib therapy. J Oncol Pharm Pract. 2017 Jun;23(4):313-317. doi: 10.1177/1078155216638551.
12. Chaucer B, Stone A, Demanes A, Seibert SM. Nivolumab-Induced Encephalitis in Hereditary Leiomyomatosis and Renal Cell Cancer Syndrome. Case Rep Oncol Med. 2018. 2018:4273231. doi: 10.1155/2018/4273231.
13. Choe JH, Andonian BJ, Kim GJ, Salama AKS. Autoimmune meningoencephalitis in a melanoma patient treated with ipilimumab. Immunotherapy. 2016. 8(10):1163-7. doi: 10.2217/imt-2016-0058.
14. Chung M, Jaffer M, Verma N, Mokhtari S, Ramsakal A, Peguero E. Immune checkpoint inhibitor induced anti-glutamic acid decarboxylase 65 (Anti-GAD 65) limbic encephalitis responsive to intravenous immunoglobulin and plasma exchange. J Neurol. 2020. 267:1023–1025. doi: 10.1007/s00415-019-09666-6.
15. Cordes LM, Davarpanah NN, Reoma LB, Gasmi B, Quezado M, Khan OI, et al. Neurotoxicities associated with checkpoint inhibitors: Two case reports and a review of the literature. Clin Case Reports. 2020. 8:24–32. doi: 10.1002/ccr3.2534.
16. De la Hoz A, Foolad F, Gallegos C, Kornblau S, Kontoyiannis DP. Nivolumab–induced encephalitis post allogeneic stem cell transplant in a patient with Hodgkin’s disease. Bone Marrow Transplant. 2019. 4(5):749-751. doi: 10.1038/s41409-018-0363-6.
17. Desforges P, Esfahani K, Bouganim N. Programmed Cell Death Ligand 1–Induced Coma From Diffuse Cerebritis. J Oncol Pract. 2018. 14(2):134-135. doi: 10.1200/JOP.2017.024992.
18. Di Stefano AL, Savatovsky J, Feuvret L, Villa C, Reina V, Pha M, et al. CNS inflammatory disorder after concurrent radiotherapy-temozolomide and nivolumab in a glioblastoma patient. Neuro. Oncol. 2019. 21(1):139-141. doi: 10.1093/neuonc/noy168.
19. Du Rusquec P, Peyre A, Toulgoat F, Honnorat J, Raimbourg J. Fatal Anti-Ma2 Encephalitis Related to Treatment of Malignant Pleural Mesothelioma With a Combination of Anti-Programmed Death 1 and Anti–Cytotoxic T-Lymphocyte Associated Protein 4 Antibodies. J. Thorac. Oncol. 2019. p. e174–176. doi: 10.1016/j.jtho.2019.03.017.
20. Elkayam N, Sharma S. Dual checkpoint inhibitor induced autoimmune encephalitis. Arch Oncol. 2019;25:22–24.
21. Erol-Yıldız R, Kızılay T, Tüzün E, Mısırlı H, Türkoğlu R. Nivolumab-induced autoantibody negative limbic encephalitis in a patient with Hodgkin lymphoma. Leuk. Lymphoma. 2020. p. 1519–1521. doi: 10.1080/10428194.2020.1725508.
22. Fellner A, Makranz C, Lotem M, Bokstein F, Taliansky A, Rosenberg S, et al. Neurologic complications of immune checkpoint inhibitors. J Neurooncol. 2018. 137(3):601-609. doi: 10.1007/s11060-018-2752-5.
23. Feng S, Coward J, McCaffrey E, Coucher J, Kalokerinos P, O’Byrne K. Pembrolizumab-Induced Encephalopathy: A Review of Neurological Toxicities with Immune Checkpoint Inhibitors. J. Thorac. Oncol. 2017. 12(11):1626-1635. doi: 10.1016/j.jtho.2017.08.007.
24. Freitas C, Fernandes G. EP1.04-04 Pembrolizumab-Induced Fatal Encephalopathy. J Thorac Oncol. 2019;14:S967–S968. <https://doi.org/10.1016/j/jtho.2019.08.2120>.
25. Fujiwara S, Mimura N, Yoshimura H, Fujimoto D, Ito M, Mori R, et al. Elevated adenosine deaminase levels in the cerebrospinal fluid in immune checkpoint inhibitor-induced autoimmune encephalitis. Intern Med. 2019. 58(19):2871-2874. doi: 10.2169/internalmedicine.2537-18.
26. Galmiche S, Lheure C, Kramkimel N, Franck N, Boitier F, Dupin N, et al. Encephalitis induced by immune checkpoint inhibitors in metastatic melanoma: a monocentric retrospective study. J. Eur. Acad. Dermatology Venereol. 2019. 33(12):e440-e443. doi: 10.1111/jdv.15756.
27. Garcia CA, El-Ali A, Rath TJ, Contis LC, Gorantla V, Drappatz J, et al. Neurologic immune-related adverse events associated with adjuvant ipilimumab: Report of two cases. J Immunother Cancer. 2018. 6(1):83. doi: 10.1186/s40425-018-0393-z.
28. Gill AJ, Perez MA, Perrone CM, Bae CJ, Pruitt AA, Lancaster E. A case series of PD-1 inhibitor-associated paraneoplastic neurologic syndromes. J Neuroimmunol. 2019. 334:576980. doi: 10.1016/j.jneuroim.2019.576980.
29. Hottinger AF, De Micheli R, Guido V, Karampera A, Hagmann P, Du Pasquier R. Natalizumab may control immune checkpoint inhibitor-induced limbic encephalitis. Neurol. Neuroimmunol. NeuroInflammation. 2018. 5(2):e439. doi: 10.1212/NXI.0000000000000439.
30. Gkoufa A, Gogas H, Diamantopoulos PT, Ziogas DC, Psichogiou M. Encephalitis in a Patient With Melanoma Treated With Immune Checkpoint Inhibitors: Case Presentation and Review of the Literature. J Immunother. 2020. 43(7):224-229. doi: 10.1097/CJI.0000000000000326.
31. Guidi, A., Violati, M, Blasi, M., Ferrari, E., Luciani, A., Codeca, C., Ferrari, D. Autoimmune related encephalitis during treatment with nivolumab for advanced head and neck cancer: a case report. Tumori. 2020. 106(6):NP23-NP28. doi: 10.1177/0300891620951262.
32. Herlopian A., Mahmoud. F., David, R., Samant, R., Gokden, M., Hutchins, L., Ong, S. Neurological complication of ipilimumab therapy in patients with metastatic melanoma. Neurology. 2015. 84(14_supplement). [https://doi.org/10.1212/WNL.84.14 supplement.P3.136](https://doi.org/10.1212/WNL.84.14%20supplement.P3.136).
33. Hottinger AF, De Micheli R, Guido V, Karampera A, Hagmann P, Du Pasquier R. Natalizumab may control immune checkpoint inhibitor-induced limbic encephalitis. Neurol. Neuroimmunol. NeuroInflammation. 2018. 5(2):e439. doi: 10.1212/NXI.0000000000000439.
34. Hsieh AHC, Faithfull S, Brown MP. Risk of cumulative toxicity after complete melanoma response with pembrolizumab. BMJ Case Rep. 2017. 2017:bcr2016218308. doi: 10.1136/bcr-2016-218308.
35. Ito M, Fujiwara S, Fujimoto D, Mori R, Yoshimura H, Hata A, et al. Rituximab for nivolumab plus ipilimumabinduced encephalitis in a small-cell lung cancer patient. Ann. Oncol. 2017. 28(9):2318-2319. doi: 10.1093/annonc/mdx252.
36. Kadosh D, Moore JA, Shenfeld J, Mina BA. Coinciding Pneumonitis and Encephalitis After Keytruda Therapy. AJRCCM. 2020. 201:A3142. <https://doi.org/10.1164/ajrccm-conference.2020.201.1_MeetingAbstracts.A3142>.
37. Kanbour A, Rasul KI, Albader SB, Sulaiman RJ Al, Melikyan G, Farghaly H, et al. Pancytopenia and limbic encephalopathy complicating immunotherapy for clear cell endometrial cancer with microsatellite instability-high (MSI-H). Onco Targets Ther. 2019.12:9965–73. doi: 10.2147/OTT.S223616.
38. Kang K, Zheng K, Zhang Y. Paraneoplastic Encephalitis and Enteric Neuropathy Associated With Anti-Hu Antibody in a Patient Following Immune-checkpoint Inhibitor Therapy. J Immunother. 2020. 43(5):165-168. doi: 10.1097/CJI.0000000000000314.
39. Kapadia RK, Ney DE, Hannan M, Farley M, Pastula DM, Piquet AL. Glial fibrillary acidic protein (GFAP) associated autoimmune meningoencephalitis in a patient receiving nivolumab. J Neuroimmunol. 2020. 344:577259. doi: 10.1016/j.jneuroim.2020.577259.
40. Khoja L, Maurice C, Chappell M, Macmillan L, Al-Habeeb AS, Al-Faraidy N, et al. Eosinophilic fasciitis and acute encephalopathy toxicity from pembrolizumab treatment of a patient with metastatic melanoma. Cancer Immunol Res. 2016. 4(3):175-8. doi: 10.1158/2326-6066.CIR-15-0186.
41. Kim A, Keam B, Cheun H, Lee ST, Gook HS, Han MK. Immune-checkpoint-inhibitor-induced severe autoimmune encephalitis treated by steroid and intravenous immunoglobulin. J Clin Neurol. 2019. 15(2):259-261. doi: 10.3988/jcn.2019.15.2.259.
42. Kopecký J, Kubeček O, Geryk T, Slováčková B, Hoffmann P, Žiaran M, et al. Nivolumab induced encephalopathy in a man with metastatic renal cell cancer: A case report. J Med Case Rep. 2018. 12(1):262. doi: 10.1186/s13256-018-1786-9.
43. Laserna A, Tummala S, Patel N, El Hamouda DEM, Gutiérrez C. Atezolizumab-related encephalitis in the intensive care unit: Case report and review of the literature. SAGE Open Med Case Reports. 2018. 2:6:2050313X18792422. doi: 10.1177/2050313X18792422.
44. Larkin J, Chmielowski B, Lao CD, Hodi FS, Sharfman W, Weber J, Suijkerbuijk KPM, Azevedo S, Li H, Reshef D, Avila A, Reardon DA. Neurologic Serious Adverse Events Associated with Nivolumab Plus Ipilimumab or Nivolumab Alone in Advanced Melanoma, Including a Case Series of Encephalitis. Oncologist. 2017. 22(6):709-718. doi: 10.1634/theoncologist.2016-0487.
45. Leempoel J, Ruyssen A, Kessler R, Van Pesch V, Gille M. Anti-Ma2/Ta paraneoplastic rhombencephalitis in a patient with lung cancer responsive to anti-PD1 therapy. Acta Neurol. Belg. 2020. 120(2):451-452. doi: 10.1007/s13760-019-01179-3.
46. Leitinger M, Varosanec M V., Pikija S, Wass RE, Bandke D, Weis S, et al. Fatal necrotizing encephalopathy after treatment with nivolumab for squamous non-small cell lung cancer: Case report and review of the literature. Front Immunol. 2018. 9:108. doi: 10.3389/fimmu.2018.00108.
47. Levine JJ, Somer RA, Hosoya H, Squillante C. Atezolizumab-induced Encephalitis in Metastatic Bladder Cancer: A Case Report and Review of the Literature. Clin Genitourin Cancer. 2017. 15(5):e847-e849. doi: 10.1016/j.clgc.2017.03.001.
48. Lyons S, Joyce R, Moynagh P, O’Donnell L, Blazkova S, Counihan TJ. Autoimmune encephalitis associated with Ma2 antibodies and immune checkpoint inhibitor therapy. Pract Neurol. 2020. 20:256–9. doi: 10.1136/practneurol-2019-002464.
49. Mandel JJ, Olar A, Aldape KD, Tremonnt-Lukats IW. Lambrolizumab induced central nervous system (CNS) toxicity. J Neurol Sci. 2014. 344(1-2):229-31. doi: 10.1016/j.jns.2014.06.023.
50. Matsuoka H, Kimura H, Koba H, Tambo Y, Ohkura N, Hara J, et al. Nivolumab-induced Limbic Encephalitis with Anti-Hu Antibody in a Patient With Advanced Pleomorphic Carcinoma of the Lung. Clin Lung Cancer. 2018. 19(5):e597-e599.  doi: 10.1016/j.cllc.2018.04.009.
51. Maurice C, Schneider R, Kiehl TR, Bavi P, Roehrl MHA, Mason WP, et al. Subacute CNS demyelination after treatment with nivolumab for melanoma. Cancer Immunol Res. 2015. 3(12):1299-302. doi: 10.1158/2326-6066.CIR-15-0141.
52. Niki M, Nakaya A, Kurata T, Nakahama K, Yoshioka H, Kaneda T, et al. Pembrolizumab-induced autoimmune encephalitis in a patient with advanced non-small cell lung cancer: A case report. Mol Clin Oncol. 2018. 10(2):267-269. doi: 10.3892/mco.2018.1777.
53. Nowosielski M, Di Pauli F, Iglseder S, Wagner M, Hoellweger N, Nguyen VA, et al. Encephalomyeloneuritis and arthritis after treatment with immune checkpoint inhibitors. Neurol Neuroimmunol neuroinflammation. 2020. 7(4):e773. doi: 10.1212/NXI.0000000000000773.
54. Ortega SG, Frank S, Stefan D, Jakob P, Sabine G, Jörg HP, et al. Toxicity associated with PD-1 blockade after allogeneic haematopoietic cell transplantation. Swiss Med Wkly. 2019. 149:w20150. doi: 10.4414/smw.2019.20150.
55. Papadopoulos KP, Romero RS, Gonzalez G, Dix JE, Lowy I, Fury M. Anti‐Hu‐Associated Autoimmune Limbic Encephalitis in a Patient with PD‐1 Inhibitor‐Responsive Myxoid Chondrosarcoma. Oncologist. 2018. 23(1):118-120. doi: 10.1634/theoncologist.2017-0344.
56. Patel AR, Connors S, Wardak Z, Brugarolas J, Patel TR. Inflammatory Reaction Secondary to Immune Checkpoint Inhibitor Therapy Mimicking a Post-Operative Brain Abscess. World Neurosurg. 2019. 129:354-358. doi: 10.1016/j.wneu.2019.06.024.
57. Pillonel V, Dunet V, Hottinger AF, et al. Multiple nivolumab-induced CNS demyelination with spontaneous resolution in an asymptomatic metastatic melanoma patient. J Immunother Cancer. 2019. 7(1):336. doi: 10.1186/s40425-019-0818-3.
58. Quach HT, Robbins CJ, Balko JM, Chiu CY, Miller S, Wilson MR, et al. Severe Epididymo‐Orchitis and Encephalitis Complicating Anti‐PD‐1 Therapy. Oncologist. 2019. 24(7):872-876. doi: 10.1634/theoncologist.2018-0722.
59. Raskin J, Masrori P, Cant A, Snoeckx A, Hiddinga B, Kohl S, et al. Recurrent dysphasia due to nivolumab-induced encephalopathy with presence of Hu autoantibody. Lung Cancer. 2017. 109:74-77. doi: 10.1016/j.lungcan.2017.05.002.
60. Robert L, Langner-Lemercier S, Angibaud A, Sale A, Thepault F, Corre R, et al. Immune-related Encephalitis in Two Patients Treated With Immune Checkpoint Inhibitor. Clin Lung Cancer. 2020. 21(5):e474–e477. doi: 10.1016/j.cllc.2020.03.006.
61. Richard K, Weslow J, Porcella SL, Nanjappa S. A Case Report of Steroid Responsive Nivolumab-Induced Encephalitis. Cancer Control. 2017. 24(5):1073274817729069. doi: 10.1177/1073274817729069.
62. Rotz SJ, Leino D, Szabo S, Mangino JL, Turpin BK, Pressey JG. Severe cytokine release syndrome in a patient receiving PD-1-directed therapy. Pediatr Blood Cancer. 2017. 64(12). doi: 10.1002/pbc.26642.
63. Salam, S., Lavin, T., Turan, A. Limibic encephalitis following immunotherpay against metastatic malignant melanoma. BMJ Case Rep. 2016. 2016:bcr2016215012. doi: 10.1136/bcr-2016-215012.
64. Santomasso, B., D’Angelo, S. Anti-CRMP5 associated paraneoplastic neurologic syndrome developing in a patient with metastatic merkel cell carcinoma during immune checkpoint inhibitor treatment. Neurology. 2018. 90(15_supplement). https://doi.org/10.1212/WNL.90.15_supplement.P5.409.
65. Sanchis-Borja M, Ricordel C, Chiappa AM, Hureaux J, Odier L, Jeannin G, et al. Encephalitis related to immunotherapy for lung cancer: Analysis of a multicenter cohort. Lung Cancer. 2020. 143:36–9. doi: 10.1016/j.lungcan.2020.03.006.
66. Sechi E, Markovic SN, McKeon A, Dubey D, Liewluck T, Lennon VA, et al. Neurologic autoimmunity and immune checkpoint inhibitors: Autoantibody profiles and outcomes. Neurology. 2020. 95:e2442–52. doi: 10.1212/WNL.0000000000010632.
67. Schneider S, Potthast S, Komminoth P, Schwegler G, Böhm S. PD-1 Checkpoint Inhibitor Associated Autoimmune Encephalitis. Case Rep Oncol. 2017. 10(2):473-478. doi: 10.1159/000477162.
68. Shah S, Dunn-Pirio A, Luedke M, Morgenlander J, Skeen M, Eckstein C. Nivolumab-Induced Autoimmune Encephalitis in Two Patients with Lung Adenocarcinoma. Case Rep Neurol Med. 2018. 2018:2548528. doi: 10.1155/2018/2548528.
69. Shah N, Jacob J, Househ Z, Shiner E, Baird L, Soudy H. Unchecked immunity: A unique case of sequential immune-related adverse events with Pembrolizumab. J Immunother Cancer. 2019. 7(1):247. doi: 10.1186/s40425-019-0727-5.
70. Sherry AD, Bezzerides M, Khattab MH, Luo G, Ancell KK, Kirschner AN. An autoimmune-based, paraneoplastic neurologic syndrome following checkpoint inhibition and concurrent radiotherapy for merkel cell carcinoma: case report. Strahlentherapie und Onkol. 2020.196:664–670. doi: 10.1007/s00066-020-01582-3.
71. Shibaki R, Murakami S, Oki K, Ohe Y. Nivolumab-induced autoimmune encephalitis in an anti-neuronal autoantibody-positive patient. Jpn J Clin Oncol. 2019. 49(8):793-794. doi: 10.1093/jjco/hyz087.
72. Stein MK, Summers BB, Wong CA, Box HL, Cleveland KO. Meningoencephalitis following ipilimumab administration in metastatic melanoma. Am J Med Sci. 2015. 350(6):512-3. doi: 10.1097/MAJ.0000000000000584.
73. Strik H, Keber U, Hammoud WA, Riera-Knorrenschild J, Carl B, Dodel R, et al. Immune checkpoint inhibitor–associated CNS autoimmune disorder (ICICAD) following nivolumab treatment: A new entity of drug-induced autoimmune encephalitis? Eur. J. Cancer. 2017. 87:205-208. doi: 10.1016/j.ejca.2017.09.026.
74. Tadipatri, Ramya, Tarzi, F.P. A rare case of autoimmune encephalitis in a patient after treatment of metastatic melanoma with a combination of immune checkpoint inhibitors nivolumab and ipilimumab. Neurology. 2020. 94(15_supplement). <https://doi.org/10.1212/WNL.94.15_supplement.447>.
75. Taillefer VT, Pigeon M, Chen M, Larochelle C, Florescu M, Bélanger K, et al. Very high-dose methylprednisolone for treatment of nivolumab-induced limbic encephalitis: A case report. J Oncol Pharm Pract. 2020. 26:1538–43. doi: 10.1177/1078155220904147.
76. Tatsumi S, Uryu K, Iwasaki S, Harada H. A Case of Anti-CRMP5 Paraneoplastic Neurological Syndrome Induced by Atezolizumab for Small Cell Lung Cancer. Intern Med. Internal Medicine. 2020. doi: 10.2169/internalmedicine.4889-20.
77. Tchapyjnikov D, Borst AJ. Immune-related Neurological Symptoms in an Adolescent Patient Receiving the Checkpoint Inhibitor Nivolumab. J Immunother. 2017. 40(7):286-288. doi: 10.1097/CJI.0000000000000177.
78. Thummalapalli R, Sena LA, Probasco JC, Gladstone DE. Checkpoint inhibitor-induced autoimmune encephalitis reversed by rituximab after allogeneic bone marrow transplant in a patient with Hodgkin lymphoma. Leuk. Lymphoma. 2020. 61(1):228-230. doi: 10.1080/10428194.2019.1658104.
79. Vogrig A, Fouret M, Joubert B, Picard G, Rogemond V, Pinto AL, et al. Increased frequency of anti-Ma2 encephalitis associated with immune checkpoint inhibitors. Neurol Neuroimmunol NeuroInflammation. 2019. 6(6):e604. doi: 10.1212/NXI.0000000000000604.
80. Vogrig A, Muniz-Castrillo S, Joubert B, Picard G, Rogemond V, Marchal C, Chiappa AM, Chanson E, Skowron F, Leblanc A, Ducray F, Honnorat J. Central nervous system complications associated with immune checkpoint inhibitors. J Neurol Neurosurg Psychiatry. 2020. 91:772-778. DOI:10.1136/jnnp-2020-323055.
81. Wang AY, Kline J, Stock W, Kosuri S, Artz A, Larson RA, et al. Unexpected Toxicities When Nivolumab Was Given as Maintenance Therapy following Allogeneic Stem Cell Transplantation: Toxicities with Maintenance Nivolumab after Allogeneic Stem Cell Transplantation. Biol Blood Marrow Transplant. 2020. 26:1025–1027. doi: 10.1016/j.bbmt.2020.01.021.
82. Williams TJ, Benavides DR, Patrice KA, Dalmau JO, De Ávila ALR, Le DT, et al. Association of autoimmune encephalitis with combined immune checkpoint inhibitor treatment for metastatic cancer. JAMA Neurol. 2016. 73(8):928-33. doi: 10.1001/jamaneurol.2016.1399.
83. Yamaguchi Y, Nagasawa H, Katagiri Y, Wada M. Atezolizumab-associated encephalitis in metastatic lung adenocarcinoma: a case report. J Med Case Rep. 2020. 14(1):88. doi: 10.1186/s13256-020-02411-y.
84. Zafar Z, Vogler C, Hudali T, Bhattarai M. Nivolumab-associated acute demyelinating encephalitis: A case report and literature review. Clin Med Res. 2019. 17(1-2):29-33. doi: 10.3121/cmr.2019.1417.
85. Zurko J, Mehta A. Association of Immune-Mediated Cerebellitis With Immune Checkpoint Inhibitor Therapy. Mayo Clin Proc Innov Qual Outcomes. 2018. 2(1):74-77. doi: 10.1016/j.mayocpiqo.2017.12.001.
